# A Magnetic-Field-Assisted Milli-Scale Robotic Assembly Machine: An Approach to Parallel Robotic Automation Systems

**DOI:** 10.3390/mi9040144

**Published:** 2018-03-23

**Authors:** Yan Liu, Nuggehalli M. Ravindra

**Affiliations:** 1 Interdisciplinary Program in Materials Science & Engineering, New Jersey Institute of Technology, 323 Dr Martin Luther King Jr Blvd, Newark, NJ 07102, USA; tcliuyan@gmail.com; 2 New Jersey Institute of Technology, 323 Dr Martin Luther King Jr Blvd, Newark, NJ 07102, USA

**Keywords:** magnetic field, assembly machine, milli-tweezer, parallel, swarm robotic systems

## Abstract

Utilizing large numbers of microrobots to heterogeneously integrate small devices to build advanced structures has long been a goal in the field of manufacturing automation. In this paper, we demonstrate a novel milli-scale robotic assembly machine with highly parallel capabilities and assisted with a programmable magnetic field. The prototype machine consists of a 16 × 16 array of electromagnets. Using this machine, we have successfully demonstrated the manipulation of up to nine milli-scale robots simultaneously. Moreover, two microrobots have been operated to demonstrate the proof of concept of two simultaneous pick-and-place light-emitting diodes (LEDs). The design and modeling of the microrobots is discussed.

## 1. Introduction

Highly parallel, automated manufacturing systems that can build macroscopic products by heterogeneous assembly of many small devices will have a major impact in several areas of manufacturing and might revolutionize the way products are manufactured.

Perhaps, the most commercially available micro-assembly technique is pick-and-place die bonding, which is widely used in printed circuit board assembly, also known as surface mount technology (SMT). However, the typical size of the surface mount devices (SMDs) handled by the SMT machine is ~1 mm, while the size of SMT robots itself is usually 1 m. There is an inherent problem in this technology, in addition to the size difference between the pick-and-place machine and the micro-device: it is a serial process. One macroscopic machine can only assemble one micro-device at a time. This has significant implications on costs, space, time, and energy, particularly when there are millions, if not billions, of small parts that need to be assembled. By contrast, nature provides numerous examples of parallel micro-assembly, such as termite mounds, which can grow to more than 7 m [1].

Various attempts have been made to build a parallel micro-assembly system, with two different methodologies. One of the methodologies is to use external forces to deliver and/or anchor micro-devices directly to receptor locations. Based on the external forces being used, these assembly techniques can be classified into four categories: (1) fluidic shape-directed self-assembly [2]; (2) capillary-driven self-assembly [3,4,5,6,7,8]; (3) electrostatically driven self-assembly [9,10]; (4) magnetically assisted self-assembly [11]. These assisted self-assembly techniques usually have specific requirements for the micro components, i.e., to be submerged in liquid, uniquely shaped, or magnetically active. Therefore, such assembly techniques have limited applications.

Another methodology is solely to overcome these limitations, which is to build robot swarms to heterogeneously assemble a massive number of small devices [12,13]. In this approach, one challenge is that, when scaling down robots, the mechanics of the microrobots are dominated by micro-scale physics, primarily due to their increasing surface-to-volume ratio, surface properties, forces, and the chemistry that becomes significant. These micro-scale forces have several different characteristics compared to that at the macro scale [14]. In addition, besides mobility and actuation, a microrobot may need power, sensors, and communication devices onboard to maximize robot capabilities; these factors make it difficult to fabricate such robots, which poses another major challenge. While the capabilities of a single robot are still very limited, microrobots may need to cooperate in a group, or simply need to avoid collisions between each other, which brings yet another major challenge, that of organizing the behavior of “swarm microrobots” or “swarm intelligence” [15].

Various works have been reported in the literature to address these challenges. Ronald S. Fearing at UC Berkeley developed a planar milli-robot system using air bearing to levitate a robot and a electromagnet coil array for robot movement [16]. Bruce R. Donald at Duke University and coworkers reported a parallel microrobotic assembly scheme using micro-electro-mechanical system (MEMS) microrobots; electrostatic force was used as single global control. An average docking accuracy of 5 µm and final assemblies with a shape match of 96% by area was reported [17]. Chytra Pawashe and coworkers at Carnegie Mellon University presented another multiple magnetic microrobot control that is achieved by an array of addressable electrostatic anchoring; magnetic robots were driven by pulsed external magnetic fields. Yet, device assembly by robots was not reported in their paper.

Perhaps, the most successful parallel robotic assembly system, involving continuous work on automated 2D micro-assembly, using diamagnetically levitated milli-robots, at Stanford Research Institute (SRI) International, has been led by Ronald E. Pelrine [18,19,20,21,22]. The machine, the DiaMagnetic Micro Manipulator (DM3, SRI International, Menlo Park, CA, USA) system, is used to control many small robots. The robots are diamagnetically levitated and driven by traces in printed circuit boards. The DM3 system uses multilayer traces and one layer of diamagnetic graphite to move the robot in a manner of a linear stepper motor. The DM3 robots are made by an array of millimeter-sized neodymium-iron-boron (NdFeB) magnets. Ronald E. Pelrine and coworkers have reported exceptional open loop repeatability of motion (200 nm rms) and relative speeds of 37.5 cm/s. A system using 130 microrobots, as small as 1.7 mm, with densities up to 12.5 robots/cm^2^ has been demonstrated [19]. A 29-cm-long cubic truss has been built using their DM3 system [23].

Despite the exceptional work at SRI and other research laboratories, robots with active tweezer arms while they are untethered, using magnetic fields, have not been reported in the literature. In addition, the robots discussed earlier, either levitated or not, are manipulated by some kind of surface and are not capable of motion under the surface or under the ceiling of the electromagnet array, or upside down. This is particularly important since, most of the time, small devices are needed to be assembled onto a substrate and the substrate cannot be used to manipulate robots at the same time. In this paper, we present a novel robotic assembly machine with parallel control of milli-scale robots running under a ceiling of an electromagnet array with an active tweezer.

## 2. System Description

### 2.1. Introduction to Magnetic-Field-Assisted Assembly (MFAA)

Our research group, at New Jersey Institute of Technology (NJIT), has been working on magnetic-field-assisted assembly (MFAA) [24,25,26,27,28] for the past 15 years. MFAA is a technique that is similar to magnetic-field-assisted statistical assembly for the integration of microstructures onto silicon or other semiconductor wafers [11]. It is proposed as a low-cost, efficient, and reliable direct assembly technique that does not rely on statistical randomness [24].

**Figure 1:**
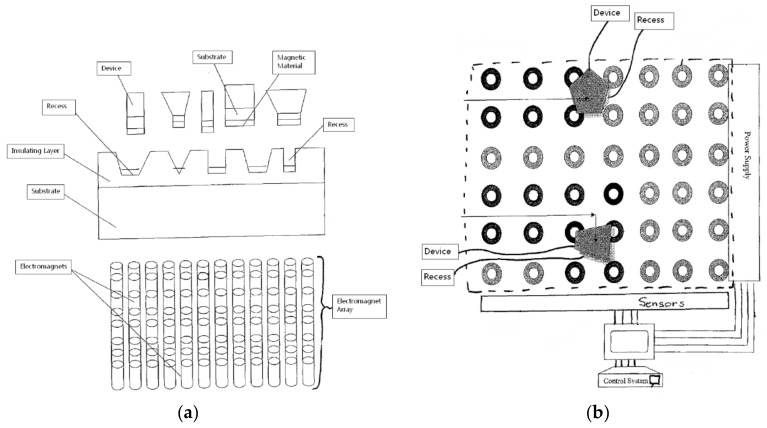
Method of magnetic field assisted self-assembly. (**a**) A schematic of the magnetic-field-assisted assembly (MFAA) method of integrating micro-components and integrated circuits. (**b**) During assembly, a moving magnetic field is applied on the back of the substrate. Reproduced with permission from [25].

Referring to Figure 1a [25], MFAA begins with the separate preparation of the substrate and micro-components. The substrate can be made from various materials, including glass, plastic, and silicon, depending on the desired application. For the integration of optoelectronics and MEMS devices with silicon integrated circuits, the starting substrate is an insulator, a semi-processed wafer, or a final wafer that contains the required integrated circuitry. In all cases, recesses are patterned either into the dielectric layer covering the wafer surface or into the surface of the insulator. The recesses are formed on the surface of the substrate in such a way that the shape and depth of the recesses match the shape and thickness of the micro-components. A highly coercive ferromagnetic material, such as cobalt, nickel, cobalt–palladium, or a cobalt–platinum alloy, is deposited on the insulator substrate or wafer. The layer is patterned to form either simple or complex features at the bottom of the recesses and is subsequently magnetized to act as a host for the micro-components. During assembly, a moving magnetic field is applied on the back of the substrate; the micro components are then fixed in place as shown in Figure 1b [25].

### 2.2. Design and Fabrication of Robot Drive System

**Figure 2:**
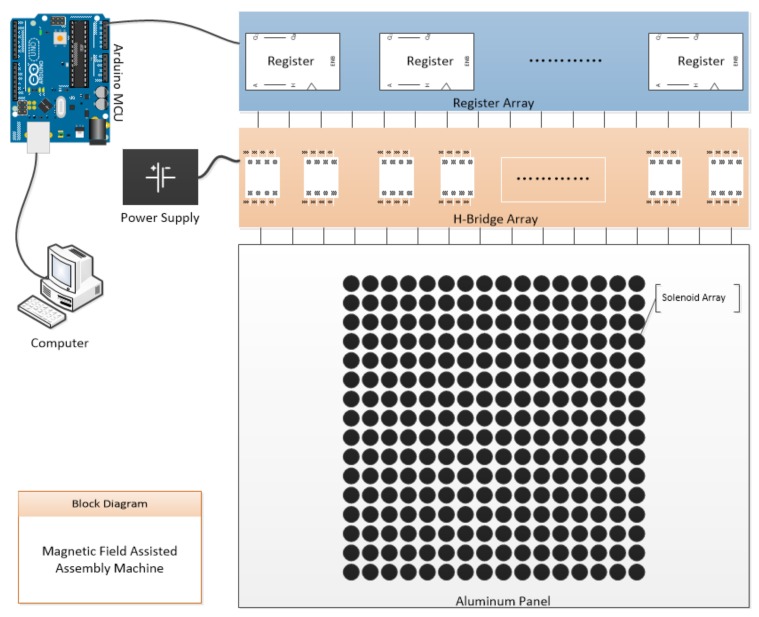
System architecture.

**Figure 3:**
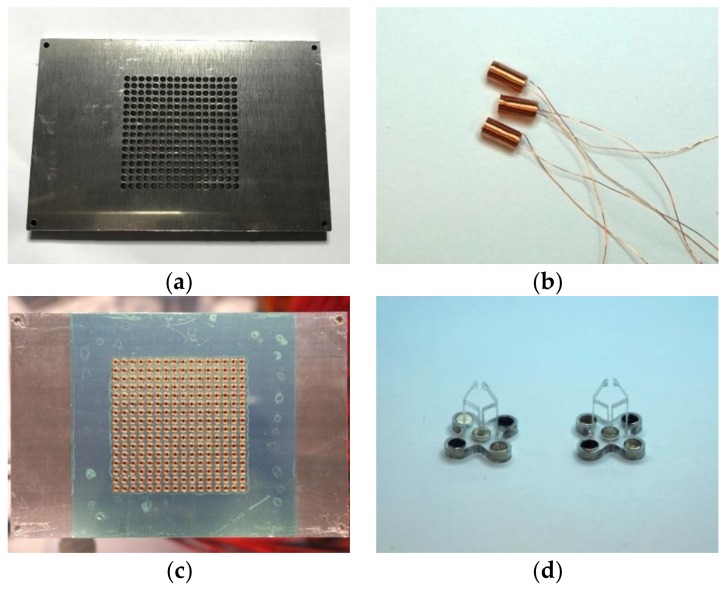
Key components of the proposed machine. (**a**) An aluminum panel milled by a computer numerical control (CNC) machine to hold small electromagnet solenoids. (**b**) Samples of electromagnet solenoid. The electromagnets are 5 mm high, with inner diameters of 1.1 mm and outer diameters of 2.5 mm. (**c**) Assembled electromagnet array panel. A thin layer of polyester tape (green color) was applied on the top of the panel. (**d**) Robot with a polycarbonate chassis and five NdFeB magnets located at four corners and the center of the chassis. A tweezer is vertically inserted and glued to each chassis.

As the magnetic-field-assisted assembly relies on a programmable magnetic field, we have built a two-dimensional array of electromagnets. The system architecture is shown in Figure 2. The array consists of two parts: an aluminum panel milled by a computer numerical control (CNC) machine and small electro magnets, as shown in Figure 3a,b.

The panel is made from a 6.35-mm-thick T6061 aluminum sheet, with dimensions of 120 mm × 80 mm. Aluminum, as a paramagnetic material, usually considered as a non-magnetic material due to its low magnetic susceptibility of 1.65 × 10^−5^ cm^3^/mole [29], is very easy to manufacture and is consequently machined. An array of 16 × 16 holes was milled on the aluminum panel by a CNC machine; all the holes were 2.55 mm in diameter, and the pitch between the holes was set to 3.0 mm. Thus, an array of 256 holes with a dimension of 48mm × 48 mm was created to hold 256 small electromagnets. The electromagnets were formed by winding seven layers of an insulated copper wire 0.1 mm in diameter, and each layer consists of 40 turns, resulting in a total of 280 turns. The electromagnets are 5 mm high with inner diameters of 1.1 mm and outer diameters of 2.5 mm. All 256 electromagnets were carefully assembled onto the aluminum panel and affixed with Loctite super glue. The assembled electromagnet array is as shown in Figure 3c. A thin layer of polyester tape (green color) with a 50 µm (25 µm polyester and 25 µm silicone adhesive) thickness was applied on the top of the panel to protect from wear and reduce friction between the panel and the robots.

For a coil with current I, ideally the magnetic field can be obtained using the Biot–Savart Law:

(1) $$\vec{B}\left(\vec{r}\right)=\mu_{0}H=\frac{\mu_{0}I}{4\pi }\int_{l}^{}\frac{d\vec{l}\times \vec{r}}{{\left|\vec{r}\right|}^{3}}$$

where $\mu_{0}=4\pi \times 10^{-7}N/A^{2}$ is the permeability of free space, and $\vec{r}$ is the vector from the current element $Id\vec{l}$ to the calculation point. For a permanent magnet with constant magnetization $\vec{M}=M_{0}\vec{z}$, where $\vec{z}$ is the direction out of the plane of the electromagnet array and perpendicular to the array. The magnetic force per unit volume between magnet and coil can be written as [30]

(2) $$d\vec{F}=\nabla \left(\vec{M}\cdot \vec{B}\right)=M_{0}\nabla B_{z}$$

where $B_{z}$ is the z component of the magnetic field of the coil. The total force is obtained from

(3) $$\vec{F}=\int_{V_{0}}^{}d\vec{F}dV=\int_{V_{0}}^{}M_{0}\nabla B_{z}dV$$

where $V_{0}$ is the volume of the permanent magnet. The vertical force $F_{z}$ will hold/anchor the permanent magnet onto the coil if the force is great enough, preventing it from falling down, even while the entire machine is turned upside down; when the current direction is switched, the force can push the magnet away and can be used for actuation.

The horizontal force can be obtained by

(4) $$F_{x}=\int_{V_{0}}^{}\mu_{0}M_{0}\frac{\partial H}{\partial x}dV$$

Given the cylindrical coil and magnet, which are symmetrical around the z direction, the magnet will be self-aligned to the coil to minimize horizontal force. If the magnet is not aligned to the coil, for example, if the magnet is at a neighboring coil location, it will be dragged toward the current coil and aligned. Therefore, with proper handling and manipulating the electromagnet array, a robot built with a permanent magnet array can be dragged to another location or can be rotated along a certain point.

An Arduino Uno R3 board was used to control the electromagnet array. The Arduino board is equipped with an ATMEGA328p microcontroller (Microchip Technology Inc., Chandler, AZ, USA) [31]. The electromagnets were driven by 256 L9110h (LG Semico, Seoul, S.Korea—information unavailable) [32] H-Bridges, which is controlled by the Arduino board. An H-bridge is an electronic circuit that enables a voltage to be applied across a load in either direction. These circuits are often used in robotics and other applications to allow direct current (DC) motors to run forwards and backwards. Each H-Bridge integrated circuit (IC) drives one solenoid; therefore, we were able to switch the direction of the current through the solenoid and hence switch the polarity of the magnetic field. Using the microcontroller, we were able to fully program the electromagnet array to generate the desired magnetic field distribution and change the field distribution very quickly. The structure of the system is shown in Figure 2.

The robot was designed using a 3 × 3 electromagnet array to operate and consisted of two parts: a polycarbonate chassis and five grade N42 NdFeB permanent magnets located at four corners and the center of the chassis, a tweezer is vertically inserted and glued to the chassis, as shown in Figure 3d. The magnetic south was marked in black in all figures.

**Figure 4:**
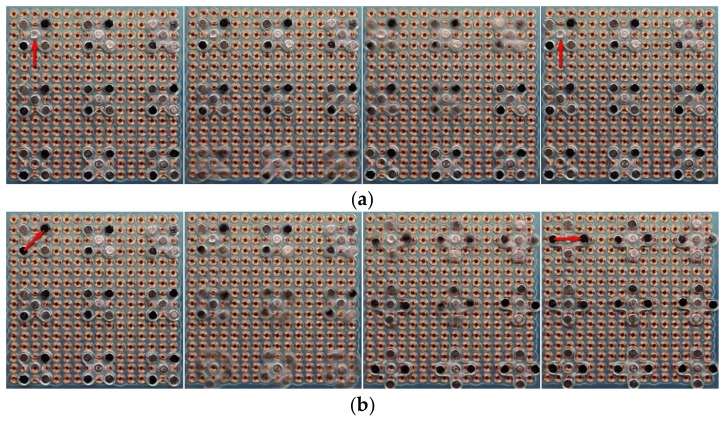
Video frames of moving robots. (**a**) Frames from Video S1 of moving nine robots to their subsequent (left) locations. (**b**) Frames from Video S1 of rotating (45°) nine robots (indicated by arrows). Tweezers have not been added to the robots here yet.

The magnetic-field-assisted assembly process requires two fundamental movements of a device: linear motion and rotation—in total, 3 degrees of freedom. Here, we demonstrate our prototype machine to be able to move and rotate nine robots simultaneously as shown in Figure 4a,b. It should be noted that we are demonstrating that all nine robots have the same movement; it does not mean that they have to move the same way. As discussed earlier, we used the same number of H-Bridges to control the electromagnets; so each electromagnet can be addressed independently, which gives us the capability of independently controlling each robot. The bottom three robots react to the change in magnetic field first while the top three robots are delayed relative to the magnetic fields; this is because the control circuit design and control signal always reaches the bottom electromagnets first. The pictures in Figure 4 are frames taken from a video. The rotating magnetic vector can be generated using an n×n electromagnet matrix. A n×n matrix will have $n^{2}$ elements with $n^{2}-{\left(n-2\right)}^{2}=4n-4$ elements in the outline of the matrix. With the magnetic field vector having 4n−4 directions, the resolution of the rotational angle will be $\frac{360}{4n-4}$. Using a 3×3 solenoid matrix will yield an angle resolution of 45°.

## 3. Milli-Robot Design and Fabrication

### 3.1. Actuation Force Measurement

**Figure 5:**
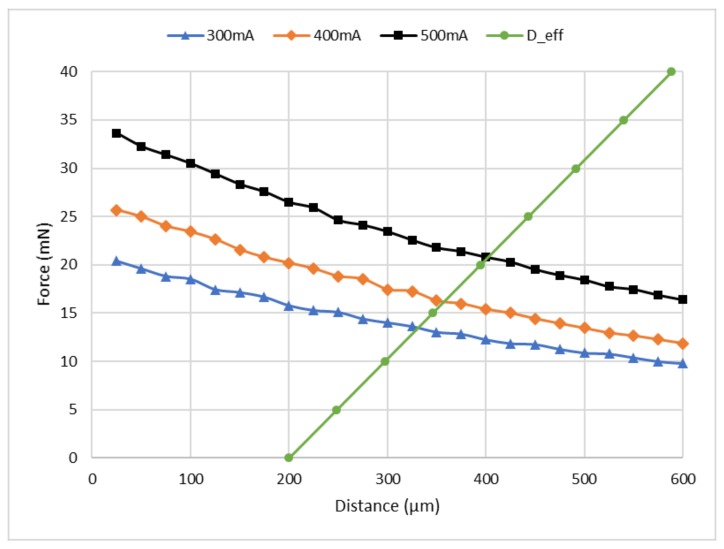
Measured results of the magnetic force vs. different currents (300 mA, 400 mA, and 500 mA) of the electromagnet coil. D_eff is the simulated curve of force load vs. effector bar displacement (with 200 μm initial distance offset).

The magnetic field utilized for robot navigation and rotation are generated by solenoids. The magnetic field acting on the NdFeB magnets will generate forces in all the directions as discussed earlier; however, in this study, we focus on the *z*-direction. This is because of the fact that we can utilize the force in the *z*-direction to actuate the tweezer as shown in Figure 4d by alternating the current through the solenoid. Additionally, the force in the *z*-direction is responsible for preventing the robot from falling down when it is moving under the ceiling of the electromagnet array. Therefore, it is critical to determine the magnitude of the force in the *z*-direction acting on the NdFeB magnets at different values of current. Hence, we set up an apparatus to experimentally test the magnetic force in the *z*-direction. The results of these measurements are shown in Figure 5. Three different currents (300 mA, 400 mA, and 500 mA) were tested for distances ranging from 25 µm to 600 µm between the permanent magnet and the electromagnet.

### 3.2. Tweezer Design and Fabrication

**Figure 6:**
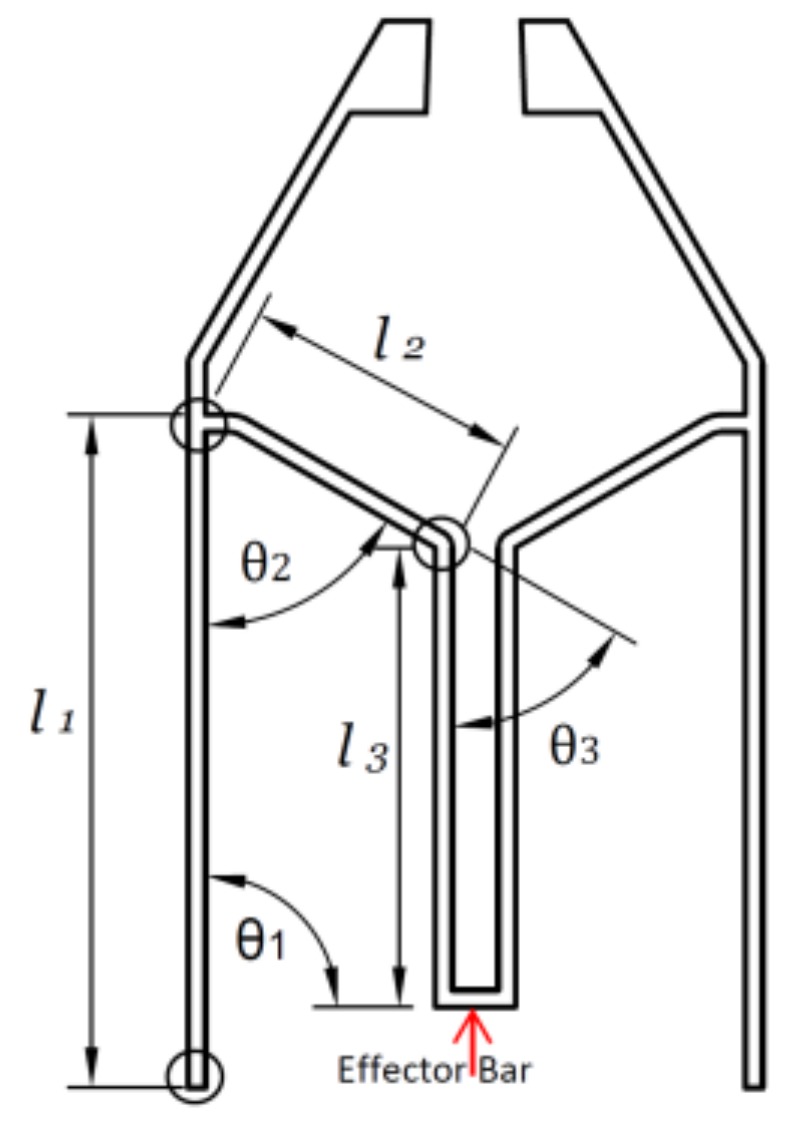
Kinematic analysis of the tweezer model using the Denavit–Hartenberg convention. The arrow indicates the effector bar of the tweezer that will be applied with load force.

In this study, we aim to make robots that can pick and place millimeter-sized devices, such as SMDs used in printed circuit boards—specifically the 0805 LEDs [33]; the 0805 LED is one of the most commonly used industrial standard LED, with dimensions of 2.0 mm × 1.25 mm × 0.8 mm (L × W × H). A model of a gripper was developed using computer-aided design (CAD) software (Autodesk Fusion 360, Autodesk, Inc., San Rafael, CA, USA) based on a four-bar linkage model [34] as shown in Figure 6. The objective is to establish the effective motion of the jaws. The Denavit–Hartenberg convention is applied for kinematics assuming perfect rigidity of each link and the free rotation of every joint around a single degree of freedom, as illustrated in Figure 6.

The homogeneous transformation matrix for the effector bar is [35]

(5) $$\left(\begin{array}{cccc} c\left(\alpha_{1}\right) & -s\left(\alpha_{1}\right) & 0 & l_{1}c\left(\theta_{1}\right)+l_{2}c\left(\alpha_{2}\right)+l_{3}s\left(\alpha_{1}\right)\\ s\left(\alpha_{1}\right) & c\left(\alpha_{1}\right) & 0 & l_{1}s\left(\theta_{1}\right)+l_{2}s\left(\alpha_{2}\right)+l_{3}c\left(\alpha_{1}\right)\\ 0 & 0 & 1 & 0\\ 0 & 0 & 0 & 1 \end{array}\right)$$

where $\alpha_{1}=\theta_{1}+\theta_{2}-\theta_{3}-\pi$ and $\alpha_{2}=\theta_{1}+\theta_{2}-\pi$, and $\theta_{3}=\theta_{1}+\theta_{2}-\frac{\pi }{2}$. The vertical displacement of effector bar becomes

(6) $$y_{\mathrm{eff}}=l_{1}\sin\left(\theta_{1}\right)-l_{2}\sin\left(\theta_{1}+\theta_{2}\right)-l_{3}$$

Given vertical link $l_{1}$, i.e., $\theta_{1}=90{}^{\circ}$, and that the tweezer is perpendicular to the robot chassis, the system becomes stiff, and the displacement of the effector bar becomes

(7) $$y_{\mathrm{eff}}=l_{1}-l_{2}\cos\left(\theta_{2}\right)-l_{3}$$

It can be observed that the vertical displacement of the effector bar is very much dependent on the angle $\theta_{2}$, and a small value of $\theta_{2}$ is desired to increase the opening range of the tweezer. This is because, as the distance between the effector and the electromagnet coil increases, the force will decrease rapidly as can be seen in Figure 5.

**Figure 7:**
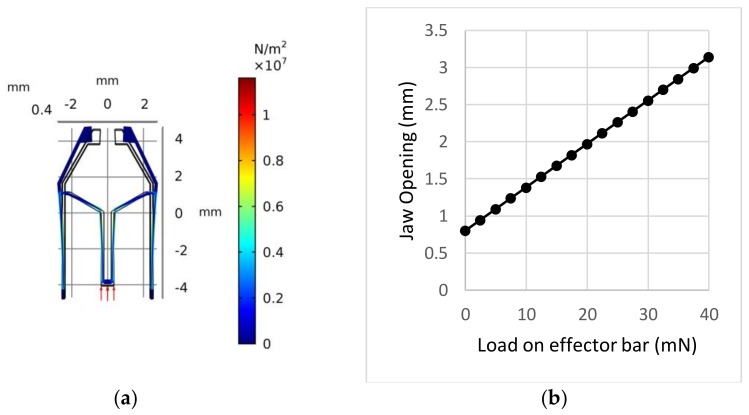
Design and simulated performance of tweezer. (**a**) Displacement field of the tweezer under force load at effector (red arrows) and von Mises stress (N/m^2^) distribution. (**b**) Simulation results of jaw opening vs. different load forces applied at the tweezer bottom effector.

Since the width of the LED is about 1.25 mm, the physical parameters of the tweezer need to be tuned so that a jaw opening greater than the width of the LED needs to be created to grip the LED. Simulations of the tweezer were conducted using finite element analysis software COMSOL Multiphysics 5.3 (COMSOL Inc., Stockholm, Sweden). The material chosen was Lexan 9034 (Total Plastics, Inc., Kalamazoo, MI, USA) polycarbonate sheet with a thickness of 1/32 in [36]; it is a clear and strong thermoplastic material with a typical tensile modulus of 238 MPa, a yield strength of 62 MPa, and a Poisson ratio of 0.37. Perfect plasticity was assumed throughout the simulation with parameters of the tweezer as follows: $l_{1}=5\;\mathrm{mm}$, $l_{2}=2\;\mathrm{mm}$, $l_{3}=4\;\mathrm{mm}$, $\theta_{2}=60{}^{\circ}$, and an initial jaw opening of 0.8 mm. The thickness of the tweezer was 1/32 in., which is the same as the thickness of the targeted material. Various tweezer wall thicknesses were tried and set to be equal to 150 μm. The results of the simulation are shown in Figure 7. As can be seen in Figure 7, with 40 mN force acting at the effector bar, the jaw will have an opening of more than 3 mm, and the maximum von Mises stress is about ~10 MPa, which is much lower than the yield strength. Thus, no irreversible plastic deformation should be expected, and the jaw opening vs. loading force is nearly linear.

**Figure 8:**
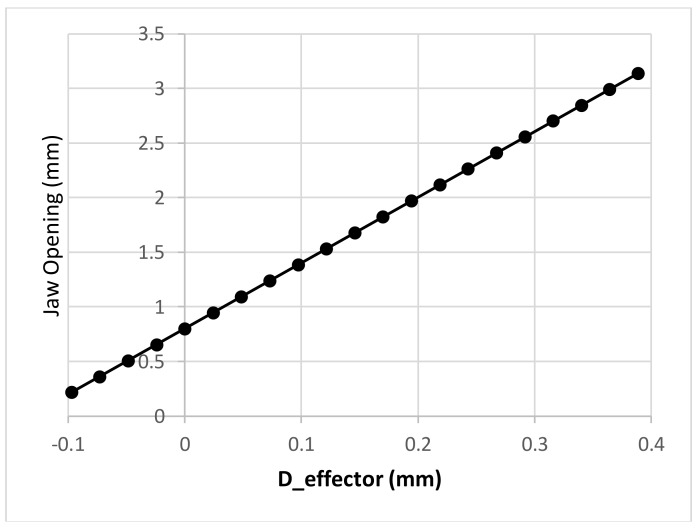
Simulation result of jaw opening vs. effector displacement. At rest, i.e., when there is no displacement in the effector, the jaw has an initial opening of 0.8 mm.

As a jaw opening of more than 1.25 mm is needed, a minimum loading force of about ~10 mN could cause an effector bar displacement of around 100 μm as can be seen in Figure 5—curve D_eff (the bar is an initial distance of 200 μm away from the electromagnet at zero force). According to our simulation result of the haw opening vs. effector displacement, shown in Figure 8, a 100 μm displacement of the effector bar should generate a jaw opening greater than 1.25 mm. Furthermore, as the typical mass of the robot is about 0.4 g, in order to manipulate the robots upside down, a minimum force of 4 mN is desired so that even one electromagnet can prevent the robot from falling down. From Figure 5, a current of 300 mA should be able to hold the robot; however, considering the anisotropy in the assembly process of all electromagnets and robots, the current was set to be 400 mA. By setting the current to 400 mA, at a distance of 350 μm between the solenoid and the magnet (which is connected with tweezer effector bar), the loading force is about 15 mN; this will create a jaw opening of around 1.6 mm (the jaw has an initial opening of 0.8 mm).

**Figure 9:**
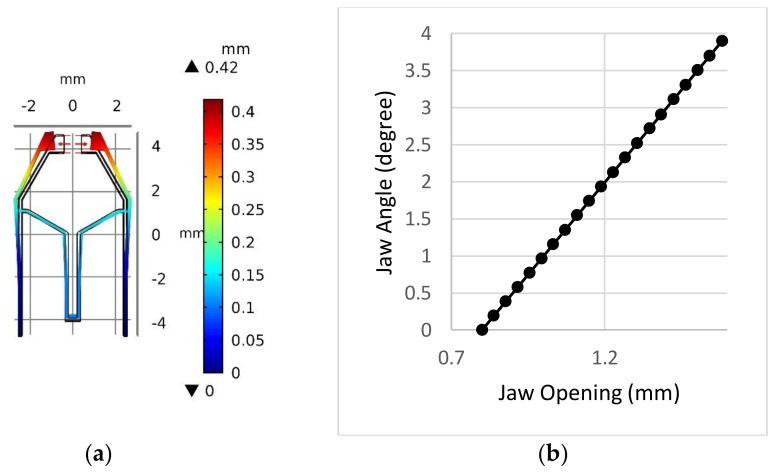
Design and simulated performance of tweezer. (**a**) Displacement field distribution of the tweezer under loading force (red arrows) at both sides of jaws. (**b**) Simulation results of jaw opening angle vs. different load forces applied at both sides of the tweezer jaw.

Since the initial jaw opening without external force is 0.8 mm, which is less than the width of the LED, i.e., 1.25 mm, a jaw displacement of 213 μm or more is needed to hold the LED. However, the displacements at the top and at the bottom of the jaw are different, as shown in Figure 9a. In order to hold the LED properly, it is important to cut a portion of the jaw so that, at a total opening of 1.25 mm, the left and right jaws of the tweezer are parallel to create uniform stress on the LED. The angle of the jaw, corresponding to the *y*-axis, has been simulated as a function of jaw displacement to determine the angle that needs to be cut, as shown in Figure 9b. The final angle was set to 1.9°.

**Figure 10:**
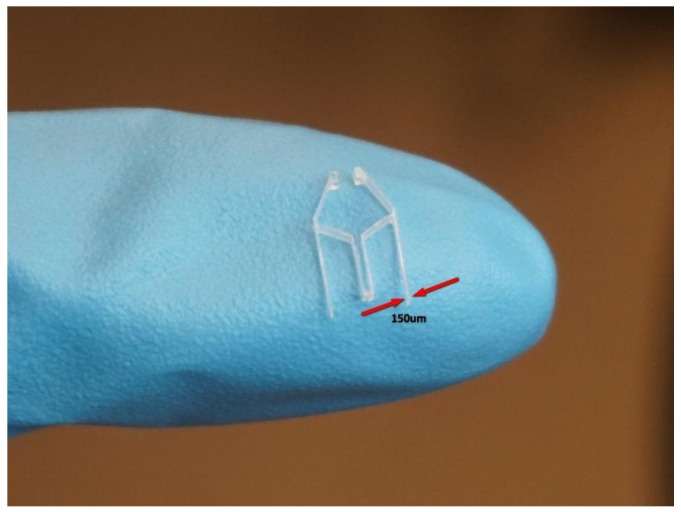
Sample tweezer fabricated using the CNC machine. The thickness of the tweezer is 0.8 mm, and the wall thickness is 150 µm.

**Figure 11:**
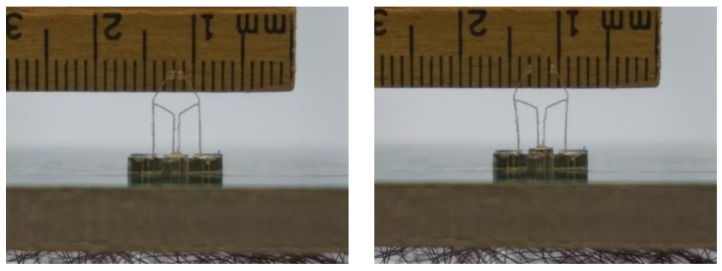
Testing the robot with the tweezer on our prototype machine. The tweezer is closed on left side of the figure and open on the right side of the figure.

The tweezers were fabricated by cutting a polycarbonate (PC) sheet 1/32 in. thick [35] using a CNC machine with a 0.016 in. flat end miniature mill, as shown in Figure 10. Tweezers were mounted to the robot chassis and tested on the electromagnetic panel. As can be seen in Figure 11, we were able to close and open the tweezer by switching the direction of the current (400 mA) in the electromagnet.

## 4. Parallel Assembly

**Figure 12:**
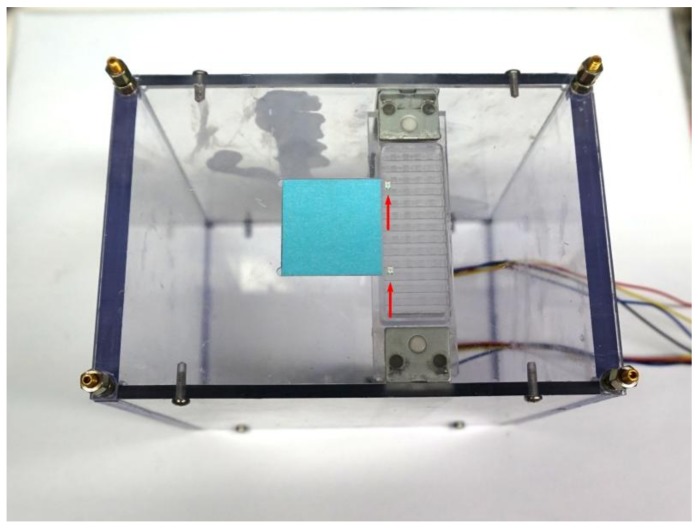
Transparent box fabricated to hold the electromagnetic panel and LED cartridge. The dimension of the box is 120 mm × 80 mm × 120 mm (L × W × H). The linear cartridge actuator is inside the box under the LED cartridge. Two LEDs (indicated by red arrows) were put into the cartridge. The box is used to hold the electromagnetic array above it by using the four bolts in the corner, and the bolts can be used to adjust the space in between.

**Figure 13:**
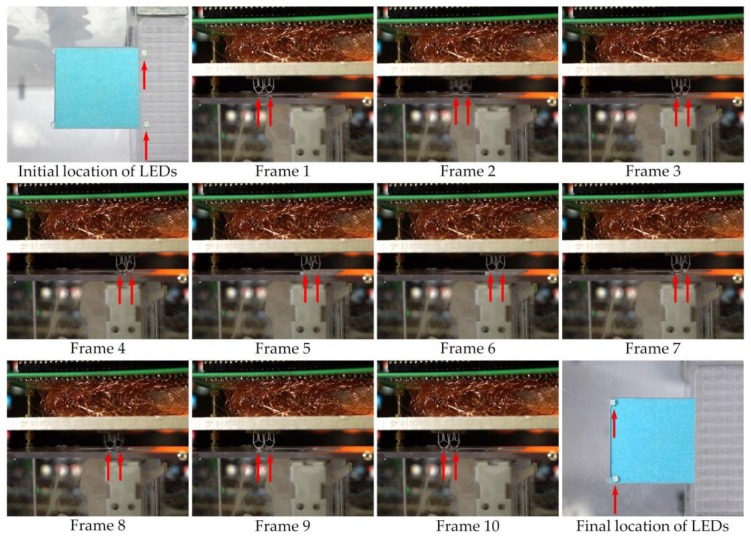
Simultaneous assembly of two LEDs by our prototype machine. The LEDs were picked-up from their initial location in the cartridge (**top left**) to their desired location on the blue sticker (**bottom right**). Others are frames taken from the demonstration assembly video (Video S2). The location of the tweezers is marked with red arrows. From left to right and from the top row to the bottom row: Initially, the LEDs reside in their initial location of the cartridge. In Frame 1, the robots are at rest. In Frame 2, robots are moving right toward the initial location of the LEDs in the cartridge. In Frame 3, the robots have arrived at the initial location of LEDs. In Frame 4, the tweezer jaws are open. In Frame 5, the cartridge moves up and the LEDs are inserted into the opening jaws. In Frame 6, the tweezer jaws are closed to hold the LEDs. In Frame 7, the cartridge move down and the LEDs are now held by the tweezers. In Frame 8, the robots are moving left toward the desired final location of the LEDs. In Frame 9, the robots have arrived at the desired location of LEDs. In Frame 10, the tweezer jaws open again to drop the LEDs. In the end, the LEDs are placed at their desired location.

A transparent box was fabricated to hold the electromagnetic panel and to hold an LED cartridge. Two LEDs were placed inside the cartridge. A 2.4 mm × 2.4 mm blue sticker as an assembly substrate was placed next to the cartridge. The sticker has double-sided tape applied on top to simulate the soldering gel used in SMT. A linear actuator below the cartridge was also controlled by the Arduino microcontroller units (MCU) to lift and drop (up and down) the cartridge, so that we could insert the LED into the tweezer jaw when it is open, as shown in Figure 12. The main purpose of the box stand is to hold the electromagnetic array above it by using the four bolts in the corner of this box. Since the robots are running upside down (tweezer toward ground) between the electromagnetic array ceiling and the substrate, there need to be some precise space between the ceiling and the substrate, and the box can be used to hold the ceiling and the bolts can be used to adjust the space in between. In order to demonstrate the proof of concept, we used two robots to simultaneously pick up the LEDs and then place them at the desired locations. The corresponding coordinates viewed via the microcontroller are from the points (11,5) and (11,12) to the locations (3,5) and (3,12) of the electromagnetic array. Therefore, each of the robots need to move eight steps right to pick up the LED and then eight steps left to drop the LED; with each step set to 300 ms, the total process took about 6 s to complete. Figure 13 consists of frames taken from the demonstration assembly video. Again, as we discussed earlier, the two robots with a tweezer are independently operated, they do not necessarily do the same work at the same time.

## 5. Conclusions

We have demonstrated a parallel assembly technique based on the controlled manipulation of magnetic-field-assisted robots. By using a 16 × 16 electromagnet array, we were able to control nine robots, about 3906 robots/m^2^; all of them can be equipped with an active tweezer. It is worth noting that our MFAA system can be easily scaled up by using a larger array of electromagnets to create a swarm robotic system. This system has the potential to assemble thousands of small devices simultaneously while the machine is kept at a conventional size (~1 m).

## 6. Patent

A patent application in support of this technology is pending.

## Supplementary Materials

The following are available online at http://www.mdpi.com/2072-666X/9/4/144/s1: Supplemental Video S1: Move and Rotate 9 Robots, Video S2: Parallel Assembly 2 LEDs.
